# Six new species of *Acomoptera* from North America (Diptera, Mycetophilidae)

**DOI:** 10.3897/zookeys.137.1764

**Published:** 2011-10-14

**Authors:** Peter H. Kerr

**Affiliations:** 1 California Department of Food and Agriculture, Plant Pest Diagnostics Branch, 3294 Meadowview Rd., Sacramento, CA, 95832–1448 USA

**Keywords:** Systematics, Sciophilinae, Sciophilini, Gnoristinae, Gnoristini, *Paratinia*, *Drepanocercus*, fungus gnats, new species

## Abstract

Six new species are described, raising the number of North American *Acomoptera* species to seven and the genus total to ten, and nearly doubling the number of species within the putative clade containing *Acomoptera*, *Drepanocercus*, and *Paratinia*. These novel species forms have implications for the concept of *Acomoptera* that in turn, may impact our understanding of its generic relationships and the evolution and composition of Gnoristinae and Sciophilinae. The new species, *Acomoptera crispa*, *Acomoptera digitata*, *Acomoptera echinosa*, *Acomoptera forculata*, *Acomoptera nelsoni*,and *Acomoptera vockerothi*, are compared with the type species of the genus, *Acomoptera plexipus* (Garrett), whose diagnostic features are imaged and illustrated for the first time. The European species, *Acomoptera difficilis* (Dziedzicki) is also illustrated and compared. *Acomoptera spinistyla* (Søli) **comb. n.** is transferred from *Drepanocercus*. A key to species is provided. Future work will seek to incorporate this knowledge into a systematic phylogenetic study of relationships between these species and their sister taxa.

## Introduction

The genus *Acomoptera* (Diptera: Mycetophilidae) was established by Vockeroth (1980) for a single Nearctic species, *Acomoptera plexipus* (Garrett) and has included only one European species, *Acomoptera difficilis* (Dziedzicki). The genus may also be present in China (Wu and Yang 1990).

By originally placing the type species in *Eudicrana*, Garrett (1925) implicitly suggested it was affiliated with the subfamily Sciophilinae (Sciophilini). Vockeroth considered *Acomoptera* a member of the Gnoristinae (Gnoristini to some authors) although he doubted its proper placement (Vockeroth 1980, 1981; Väisänen 1986). Since then, the subfamilial placement of *Acomoptera* has gone back and forth, some preferring Gnoristinae (Vockeroth 1981, Blagoderov 1998, Polevoi and Jakovlev 2004, Ševčík and Chandler 2008) while others Sciophilinae (Bechev 1999, Kjærandsen et al. 2007, Kjærandsen and Jordal 2007). Most recently, Rindal et al. (2009) recovered *Acomoptera* clustered within a paraphyletic Gnoristinae in a molecular study involving small fragments of two nuclear (18S, 28S) and one mitochondrial gene (16S).

This schizophrenic classification history for *Acomoptera* largely follows the various interpretations applied to *Acomoptera*'s putative sister taxa, *Paratinia* Mik and *Drepanocercus* Vockeroth. *Paratinia* and *Drepanocercus* have been placed either together in Sciophilinae (Bechev 1999, Kjærandsen et al. 2007), or simultaneously in Sciophilinae and Gnoristinae, respectively (Vockeroth 1980, 1981; Søli 1993, 1997; Polevoi and Jakovlev 2004). As noted by Vockeroth (1980), *Acomoptera* resembles *Paratinia* Mik in eye shape, facial and antennal structure, thoracic setation, and wing venation. Søli (1993) notes that *Paratinia* appears closely related to *Drepanocercus* on account of having nearly naked eyes, a bare proepisternum, a distinct paratergite, and a well-developed phragma; these states are also shared with *Acomoptera* (except in the last character). *Drepanocercus* and *Paratinia* are recovered as sister taxa in Søli (1997), although this study did not include *Acomoptera*. In more recent morphological studies of the family, *Drepanocercus*, *Paratinia*, and *Acomoptera* are recovered together(Chris Borkent, pers. comm.).

Before investigations into the phylogenetic relationships of *Acomoptera* and other genera proceed further, it is first important to recognize the diversity of species within *Acomoptera* itself. The holotype of the type species, *Acomoptera plexipus* (Garrett), is a female and now badly damaged and covered by fungal spores (Fig. 21B). Vockeroth (1980) associated males to the holotype by matching wing venation and proximity of collecting locality, but described the genus without illustrating the male genitalia, which are diagnostic for species. Since it was thought that only one species was present in North America, and this species was weakly characterized, *Acomoptera* collected in the region were typically assumed to be *Acomoptera plexipus*. In fact, the Nearctic supports an impressive diversity of *Acomoptera*. Recent studies in California and elsewhere in North America revealed a number of undescribed species that show a surprising degree of morphological variation, particularly in the male genitalia. These novel forms have implications for the concept of the genus and the clade including its putative sister-groups. This may in turn, also impact our understanding of the evolution and composition of two large subfamilies of Mycetophilidae, the Gnoristinae and Sciophilinae.

Here, an additional six new species are described, raising the number of North American *Acomoptera* species to seven. These new species are compared with *Acomoptera plexipus* (Garrett), whose diagnostic features are imaged and illustrated. The European species, *Acomoptera difficilis* is also illustrated and compared. A key to species of the genus is included and apparent phylogenetic affiliations between congeners are briefly discussed.

## Materials and methods

Terminology for wing venation generally follows McAlpine (1981), however interpretation of radial veins consistent with Søli(1997). Terminology for thoracic and genitalic morphology largely follows Wood (1991), McAlpine (1981), and Vockeroth (1981). Terminology of thoracic sclerites and wing veins is standardized in Figs 7 and 8, respectively. Whole specimens and genitalia were macerated in 10% KOH at approx. 95°C for 15–20 minutes to remove soft tissue, then rinsed in distilled water and dilute glacial acetic acid, and dissected in water. All genitalia preparations were placed in a drop of DMHF and dry mounted onto a small card with transparent backing, held to the pin beneath the specimen. Illustrations and plates were made using Adobe Illustrator and Adobe Photoshop Creative Suite software, aided by digital images taken using a Q-imaging Micropublisher 5.0 scope-mounted digital camera. Habitus images were taken with the same digital camera, using an LED dome lighting system (Kerr et al. 2008). Material examined includes holdings deposited in the California State Collection of Arthropods, Sacramento, California, USA (CSCA); Canadian National Collection (CNC); California Academy of Sciences, San Francisco, California, USA (CASC); and the private collection of Olavi Kurina (Tartu, Estonia). Paratypes are also deposited in 100% EtOH in the Frozen Tissue Collection of the CSCA (CSCA-FTC) at -80°C for DNA preservation. Specific collection holding and deposition information is provided in the species accounts. High resolution images of studied material are deposited and publicly available in Morphbank (http://www.morphbank.net/). Morphbank image numbers are cited within brackets, in the figure captions, and serve as embedded links.

## Taxonomy

### Acomoptera

Vockeroth

http://species-id.net/wiki/Acomoptera

Acomoptera  Vockeroth, 1980: 534

#### Type species.

 *Eudicrana plexipus* Garrett, 1925: 4, by subsequent designation.

#### Diagnosis.

This genus may be distinguished from most mycetophilid genera by the following combination of wing characters: Sc ending in C, sc-r present near middle of Sc, and R4 present and displaced from Rs by more than 2.5× its own length. *Acomoptera* may be distinguished from *Paratinia* by having wing membrane bare and from *Drepanocercus* by having the cubital fork near the level or distad of sc-r. *Acomoptera* is distinguished from *Phoenikiella* Chandler by having a setose Sc, with sc-r positioned near the middle of this vein.

#### Description.

 Body length 4.5 to 7.1 mm. Ocelli three, subequal or median ocellus reduced, lateral ocellus separated from eye margin by approximately its own diameter or clearly less. Eye with a broad distinct emargination above antennal base, with microsetae usually scattered and short, but sometimes more numerous and longer. Frons bare between ocelli and antennal bases. Frontal tubercle present. Antenna two to four times as long as thorax, 2/3rds to approximately same length as abdomen; antennal bases nearly touching, separated only by narrow tip of frontal tubercle; all flagellomeres densely setulose, elongate, flagellomere length decreases gradually toward tip. Palpus with five palpomeres; palpomere 1 short, as wide as long, remaining palpomeres longer than wide (except sometimes palpomere 2 as wide as long); palpomere 2 clearly shorter than or subequal to palpomere 3; palpomere 4 three to six times longer than wide; palpomere 5 thinner than others, six to eleven times longer than wide, usually subequal to combined length of palpomeres 3 and 4. Scutum with short appressed acrostichal and dorsocentral setae and longer and more erect sublateral setae, the intervening areas bare. Scutellum with three or four irregular rows of short to long setae, sometimes bare medially. Paratergite present; antepronotum with setae on posterior half, proepisternum bare; mesopleuron, metapleuron, and prosternum bare; metanotum with one to three erect setae laterad of base of halter; mediotergite and laterotergite bare; phragma not well developed. Wing unmarked, with dense microtrichia, without macrotrichia. Costal vein extends beyond R_5_, between approx. 0.25× and 0.33× distance between R_5_ and M_1_; sc-r crossvein present, slightly distad of middle of Sc, proximal of Rs; R4 present (when missing, teratogenic), located approx. 3× its own length from Rs, forming an elongate radial cell; medial fork approx. 3× longer than stem; cubital fork arises near level or distad of sc-r and proximad of medial fork, cubital stem shorter than CuA_1_ and approx. equal in length to or longer than CuA_2_; all cross veins bare on upper surface, all longitudinal veins setose on upper surface, except CuP and the first two sections of M bare (as an exception, *Acomoptera crispa* sp. n. may have a few setae on M_1+2_); wing veins R_1_, R_5_, M_1_, and sometimes M_2_ with at least some setae on lower surface. Hind coxa with a single vertical row of setae on at least proximal half (sometimes weak). Tibial bristles short but distinct, the longest between half and full tibial diameter in length. Anteroapical depressed area of fore tibia ovate and well developed. Tibial spur pairs of equal length, hind tibial spurs usually longer than mid tibial spurs, but sometimes mid and hind spurs subequal in length. Tarsal claws each with one or two small ventral teeth. Empodia small. Sternite 1 bare. Sternites 2 to 7 each with a pair of broad, poorly defined, submedian to sub lateral fold-lines. Terminalia not rotated. Epandrium (tergite 9) between three times as wide as long to slightly wider than long. Hypandrium (sternite 9) fused with gonocoxites, the synsclerite with or without a narrow transverse membranous area across ventral surface, with or without a ventral preapical hook-like process directed posteriorly. Gonostylus variously formed, often divided into two or three lobes. Posterodorsal process attached to the median dorsal angles of the gonocoxites variously shaped, sometimes arising as a distinctive bilobed wing-like structure whose posterior margins are darkened and toothed. Cerci broad, flat, broadly rounded apically, with fine setae. Hypoproct broad, semicircular, weakened anteromedially.

Female sternite 8 deeply emarginate posteriorly, the rest of the median area membranous or lightly sclerotized. Sternite 9 with two weakly sclerotized anterolateral areas and two slender, more heavily sclerotized submedian processes projecting posteriorly. Tergite 10 short, setose, fused on either side with sternite 10. Sternite 10 well developed, membranous medially, tapering on posterior half, posterior margin extending to apex of first segment of cercus. Cercus 2-segmented, first segment almost twice as long as wide, second segment oval.

#### Key to the Acomoptera of the world (males)

**Table:** 

1	Hook-like or fork-like hypandrial lobe present (Figs 5B, 5E, 17B, 17E, 23B, 23E)	2
–	Hypandrial lobe absent	4
2	Hypandrial lobe fork-like (Figs 16D, 17A–C)	*Acomoptera forculata* sp. n.
–	Hypandrial lobe hook-like (Figs 5B, 23B)	3
3	Hypandrial lobe long, subtending the gonostyli in part (Fig. 5E); gonostylus with club-like, ventral process that extends well beyond rest of gonostyus posteriorly (Figs 4A–C, 5A–B, 5E); Europe	*Acomoptera difficilis* Dziedzicki
–	Hypandrial lobe short, not reaching beyond posterior margin of gonocoxites (Figs 22B, 23B, 23E); gonostylus without large, posteriorly-directed club-like process; North America	*Acomoptera plexipus* Garrett
4	Gonostylus with three apical lobes of similar size and shape	*Acomoptera sinica*Wu & Yang
–	Gonostylus variously shaped, without three subequal lobes	5
5	Gonocoxites with well-developed, paired structure (‘gonocoxal comb') dorsomedially (Figs 2A, 13A, 19A, 25A)	6
–	Gonocoxites without such well-developed dorsomedial structure (e.g., Fig. 11A)	9
6	Posterior face of gonostyli with prominent cluster of curved setae dorsally (Figs 3A–B, 3D–E, 26A–B, 26D–E)	7
–	Posterior face of gonostyli without such curved setae (Figs 14A–B, 14D–E, 20A–B, 20D–E)	8
7	Gonocoxites with transverse, denticulate ridge of cuticle ventrally (Fig. 26B) ; outermost dorsal margin of gonostylus swept forward (Fig. 26D–E); ventromedial process of gonostylus usually unforked (Fig. 26E)	*Acomoptera vockerothi* sp. n.
–	Gonocoxites smooth ventrally, without transverse, denticulate ridge (Fig. 3B) ; uppermost lateral margin of gonostylus swept forward (Fig. 3D–E); ventromedial process usually forked (Fig. 3E)	*Acomoptera crispa* sp. n.
8	Inner face of gonostylus toothed dorsally (Fig. 20A)	*Acomoptera nelsoni* sp. n.
–	Inner face of gonostylus smooth dorsally (Fig. 14A)	*Acomoptera echinosa* sp. n.
9	Fork of cubital wing vein distad of base of r-m (Fig. 8); hypandrium entire (Fig. 11B)	*Acomoptera digitata* sp. n.
–	Cubital fork proximal to r-m (Ševčík 2004: fig. 1, Søli, 1993: fig. 1); hypandrium with unsclerotized area near posterior margin (Søli, 1993: fig. 2B)	*Acomoptera spinistyla* (Søli), comb. n.

### Acomoptera crispa sp. n.

urn:lsid:zoobank.org:act:30939031-FE99-43C1-B4D4-62D9FED2D02F

http://species-id.net/wiki/Acomoptera_crispa

Figs 1 [Fig F2] 3 

#### Type Material.

 Holotype: ♂, “B.C., Hixon, 21.v.1966, E.D.A. Dyer" [53.420°, –122.595°] / “HOLOTYPE 11G483 ♂ *Acomoptera crispa* Kerr 2011" [red label]. Deposited in CNC, specimen glued directly to the pin, missing ultimate two right palpomeres, missing ultimate 10 (left) and 9 (right) antennal flagellomeres, missing all legs except mid left, both wings partially torn but complete; otherwise in fair condition. Specimen dissected, male genitalia preserved in DMHF, on card marked “11G483" pinned below specimen.

#### Diagnosis.

This species is most similar to *Acomoptera vockerothi* in having similarly-shaped gonostyli that feature a line of long, curved setae near the dorsal margin. *Acomoptera crispa* may be separated from *Acomoptera vockerothi* by the gonocoxites lacking a posteromedial denticulate ridge ventrally (Figs 2B, 3B). It is also distinguished by having a longer, narrower ventromedial gonostylus lobe, which is usually forked (Fig. 3E) and the outer dorsal lobe is broadly attached laterally (Figs 2D, 3D–E).

#### Description.

 Male. Body length: 6.0 mm. Wing length: 5.8 mm.

**Figure 1.**
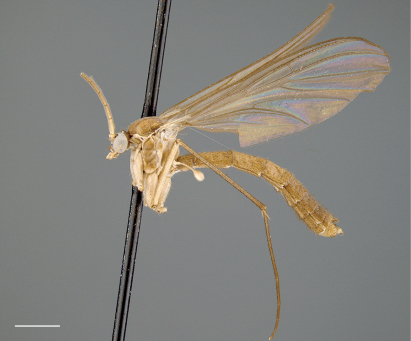
*Acomoptera crispa* sp. n., habitus, lateral view [691239]. Scale line = 1 mm.

*Coloration* (Fig. 1). Head light brown; palpomeres yellow to yellowish brown. Antennal scape and pedicel yellow to yellowish brown, flagellomeres yellowish increasingly yellowish brown toward tip. Thorax yellowish brown; area of scutum bearing acrostichal and dorsocentral setae defined by darker coloration, scutum setae gold- or golden brown-colored. Mid leg becoming increasingly brown towards tarsi; coxae yellowish or cream-colored; femur yellowish, tibia yellowish brown, tarsi brown (other legs missing, but likely having similar coloration). Wing hyaline without markings, wing veins yellowish brown; haltere stem and knob yellow to light yellowish brown. Abdominal segments concolorous, yellowish brown, with scattered yellow and brown setae. Terminalia yellowish brown.

*Head*. Ocelli slightly raised; middle ocellus approx. same size as lateral ocelli; lateral ocellus located approx. width of ocellus or less from eye margin, separated from median ocellus by approx. twice its own diameter. Eyes with sparse, inconspicuous microsetae, which are approximately as long as width of facet. Antennal length probably approx. 4.0 mm (approx. 0.8× length of abdomen). Palpus approx. 1.0–1.25× width of head (anterior view); length of palpomeres 2 and 3 subequal; palpomere 4 approx. 4.5× longer than wide; palpomere 5 approx. 10× longer than wide, subequal to or longer than combined length of palpomeres 3 and 4.

*Thorax*. Antepronotum bearing setae; remaining thoracic sclerites bare. Wing venation similar to others in the genus (e.g., *Acomoptera digitata* sp. n., Fig. 8); costal vein extends beyond R_5_, approx. 0.25× distance between R_5_ and M_1_; R_1_, R_5_, and M_1_, with at least some setae on lower surface.

**Figure 2. F2:**
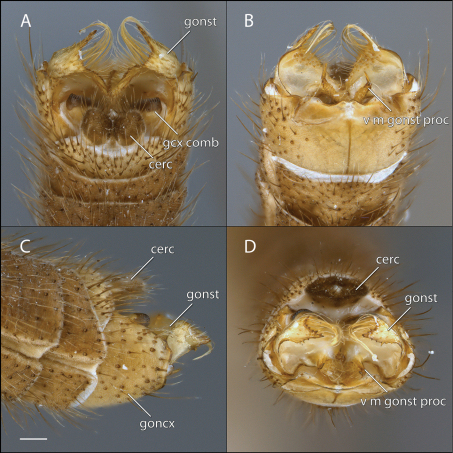
*Acomoptera crispa* sp. n., male genitalia, images: **A** dorsal view [691235] **B** ventral view [691236] **C** lateral view [691237] **D** posterior view [691237]. Scale line = 0.1 mm. Abbreviations: **cerc** cercus **epand**epandrium **gcx comb** gonocoxal comb **goncx** gonocoxites **gonst** gonostylus **v m gonst proc** ventromedial gonostylus process.

**Figure 3.**
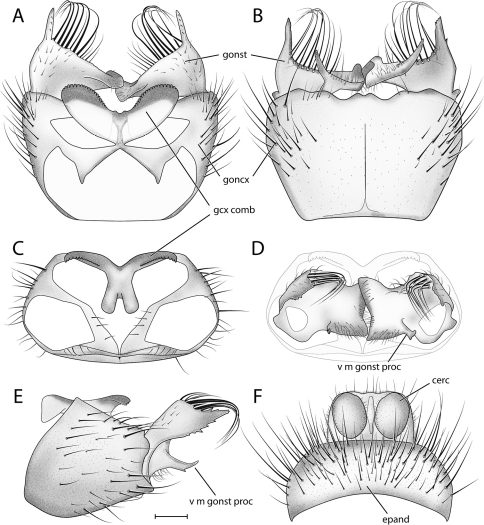
*Acomoptera crispa* sp. n., male genitalia, illustrated: **A** gonopods, dorsal view [691241] **B** gonopods, ventral view [691242] **C** gonocoxites, with gonostyli removed, posterior view [691244] **D** gonostyli, posterior view [691244] **E** gonopods, lateral view [691243] **F** epandrium, dorsal view [691240]. Scale line = 0.1 mm. Abbreviations: **cerc** cercus **epand** epandrium **gcx comb** gonocoxal comb **goncx** gonocoxites **gonst** gonostylus **v m gonst proc** ventromedial gonostylus process.

*Male Genitalia* (Figs 2–3). Epandrium approx. 3× wider than long (Fig. 3F). Gonocoxites with dorsomedial comb (Figs 3A, 3C). Gonostylus complex, as shown in Figs 2, 3A–B, 3D–E.

Female unknown.

#### Etymology.

 The species epithet “crispa" is an adjective meaning curly in Latin, and refers to the long curved setae of the gonostylus.

### Acomoptera difficilis

(Dziedzicki)

http://species-id.net/wiki/Acomoptera_difficilis

Figs 4 5

Paratinia difficilis  Dziedzicki, 1885: 169

#### Material Examined.

1 ♂, “SWEDEN: Ög: Fröåsa, 57.8833°N, 15.6666°E, N.Franc & Co 10.v–10.vi.2004, Malaise Trap" deposited in the collection of O. Kurina.

**Figure 4.**
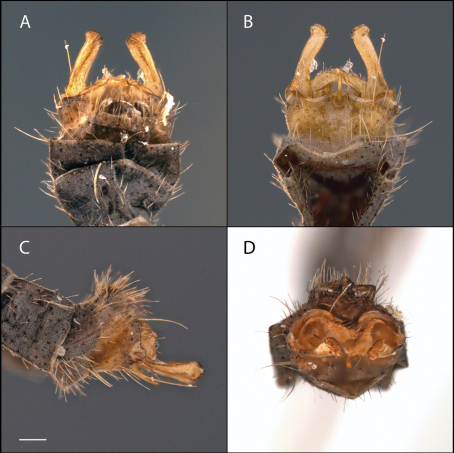
*Acomoptera difficilis*, male genitalia, images: **A** dorsal view [691256] **B** ventral view [691257] **C** lateral view [691258] **D** posterior view [691259]. Scale line = 0.1 mm.

**Figure 5.**
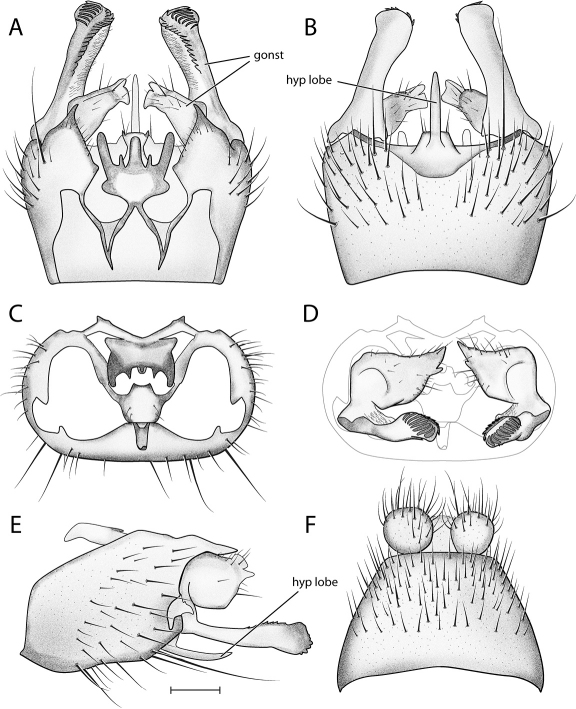
*Acomoptera difficilis*, male genitalia, illustrated: **A** gonopods, dorsal view [691261] **B** gonopods, ventral view [691262] **C** gonocoxites, with gonostyli removed, posterior view [691264] **D** gonostyli, posterior view [691265] **E** gonopods, lateral view [691263] **F** epandrium, dorsal view [691263]. Scale line = 0.1 mm. Abbreviations: **gonst** gonostylus **hyp lobe** hypandrial lobe.

### Acomoptera digitata sp. n.

urn:lsid:zoobank.org:act:593ECF9D-76E8-40E6-8C99-200FA32A64B8

http://species-id.net/wiki/Acomoptera_digitata

**Figure 6.**
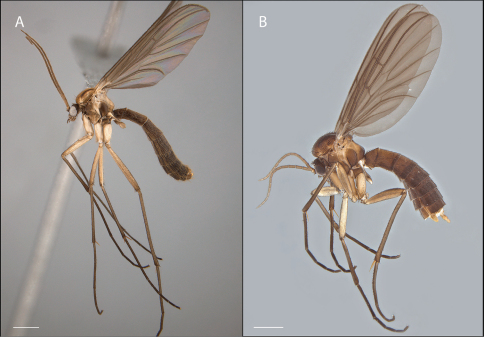
*Acomoptera digitata* sp. n., habitus, lateral view: **A** male [691271] **B** female [691264]. Scale line = 1 mm.

**Figure F8:**
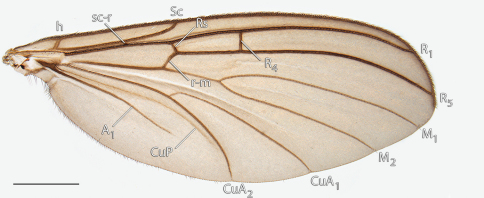
**Figure 8.** *Acomoptera digitata* sp. n., wing, dorsal view [692360]. Scale line = 1 mm.

Figs 6 [Fig F7] [Fig F8] [Fig F9] [Fig F10] 11 

#### Type Material.

 Holotype: ♂, “USA, Oregon, Curry Co., small seep (#2) on Elko road, 42°23.122'N, 124°13.736'W 925m, 2.vi.2009 G. Courtney CSCA09L472" / “HOLOTYPE 09D070 ♂ *Acomoptera digitata* Kerr 2011" [red label]. Deposited in CSCA, complete specimen in excellent condition, mounted on gray point.

Paratypes: 10 ♂♂, 1♀ “USA: CA: Del Norte Co, SixRiversNF, ForRoute16N02, nr. BearBasin Outlk, 41.8016°N, 123.7369°W, 1500masl, 3.vi-24.vii.2009 P.H.Kerr & O.Lonsdale, 6m MT, CSCA09L526" [CSCA].

#### Diagnosis.

  This species may be distinguished from all other *Acomoptera* species by the dorsoventrally elongate suboval shape of the gonostylus, which features a prominent, finger-like lobe that projects inward (Fig. 11D) and a ventral, posterior-protruding lip that bears an elongate, narrow process (Figs 11D–E).

#### Description.

 Male. Body length (n=6): 5.2–6.4 mm (avg = 5.9 mm). Wing length: 5.0–6.1 mm (avg = 5.6 mm).

**Figure 7. F7:**
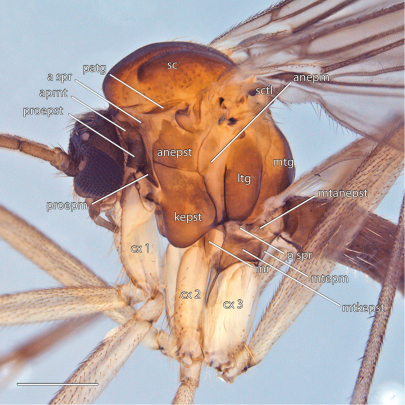
*Acomoptera digitata* sp. n., thorax, lateral view [692363]. Scale line = 0.5 mm. Abbreviations: **anepm** anepimeron **anepst** anepisternum **aprnt** antepronotum **a spr** anterior spiracle **cx** coxa **kepst** katepisternum **ltg** laterotergite **mr** meron **mtg** mediotergite **mtanepst** metanepisternum **mtepm** metepimeron **mtkepst** metakatepisternum **p spr** posterior spiracle **patg** paratergite **proepm** proepimeron **proepst** proepisternum **sc** scutum **sctl** scutellum.

*Coloration* (Figs 6A, 7). Head brown; palpomeres light brown. Antennal scape light brown, pedicel and flagellomeres yellow to yellowish brown. Thorax variously brown to cream-colored, in parts; pair of darker brown markings on scutum laterad of dorsocentral setae, scutum setae gold- or golden brown-colored; antepronotum, proepisternum, and proepimeron dark brown, anepisternum, katepisternum, and meron brown; anepimeron cream-colored and noticeably lighter in color than surrounding sclerites; laterotergite brown; metanepisternum and metakatepisternum brown to dark brown; mediotergite brown centrally, cream-colored laterally. Legs becoming increasingly brown towards tarsi, coxae cream-colored (coxal setae yellow); femur yellowish or cream-colored, tibia yellowish brown, tarsi light brown. Wing hyaline without markings, wing veins light brown; haltere stem and knob cream-colored to yellowish brown. Abdominal segments concolorous, brown, with golden brown setae. Terminalia yellowish brown to brown.

*Head*. Ocelli slightly raised; middle ocellus smaller or about the same size as lateral ocelli; lateral ocellus located approx. its own width (approx. width of two eye facets) from eye margin, separated from median ocellus by 2–3× its own diameter. Eyes with sparse, inconspicuous microsetae, which are approximately as long as width of facet. Face with brown setae, longest of which approx. same length as width of face. Antennal length approx. 0.75× length of abdomen. Palpus shorter than width of head (anterior view); palpomere 2 clearly shorter than palpomere 3; palpomere 4 approx. 3× longer than wide; palpomere 5 approx. 6× longer than wide, shorter than combined length of palpomeres 3 and 4.

*Thorax* (Fig. 7). Antepronotum bearing setae; remaining thoracic sclerites bare. Tarsal claw usually with two small ventral teeth. Wing venation as in Fig. 8; costal vein extends beyond R_5_, approx. 0.25× distance between R_5_ and M_1_; R_1_, R_5_, and M_1_ with at least some setae on lower surface.

*Male Genitalia* (Figs 10–11). Epandrium approx. 2× wider than long (Fig. 11F). Gonocoxites without developed dorsomedial comb (Figs 11A, 11C). Gonostylus complex, as shown in Figs 10, 11A–B, 11D–E.

Female. Body length (n=1): 5.9 mm; wing length (n=1): 5.6 mm.

**Figure 9. F9:**
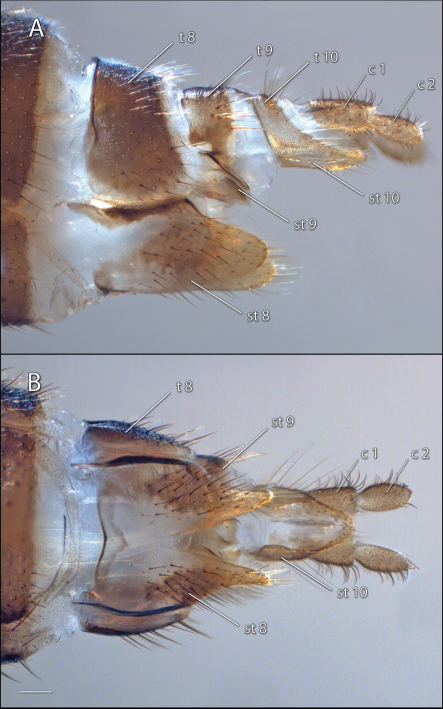
*Acomoptera digitata* sp. n., female genitalia: **A** lateral view [692365] **B** ventral view [692366]. Scale line = 0.1 mm. Abbreviations: **c** cercus **st** sternite **t** tergite.

As male in all aspects except the following:

Generally moderately darker than male (Fig. 6B). Antennal scape brown, pedicel and flagellomeres light brown, yellowish brown, or brown. Scutum setae black. Legs becoming increasingly brown towards tarsi, coxae light brown (coxal setae black); fore femur yellowish or cream-colored, mid and hind femora light brown or brown; tibia and tarsi brown to light brown. Wing membrane darker and wing veins stronger than in male; abdominal setae black. Terminalia yellowish or yellowish light brown, form as in Fig. 9.

**Figure 10. F10:**
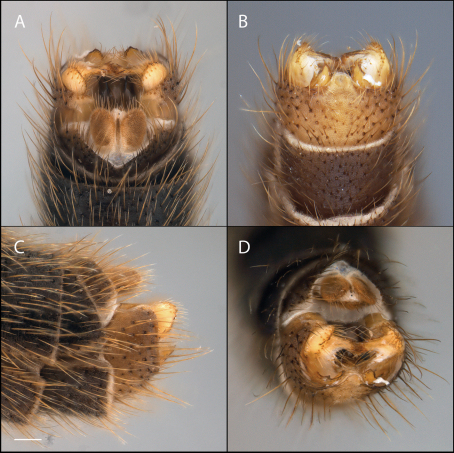
*Acomoptera digitata* sp. n., male genitalia, images: **A** dorsal view [692367] **B** ventral view [692368] **C** lateral view [692369] **D** posterior view [692370]. Scale line = 0.1 mm.

**Figure 11.**
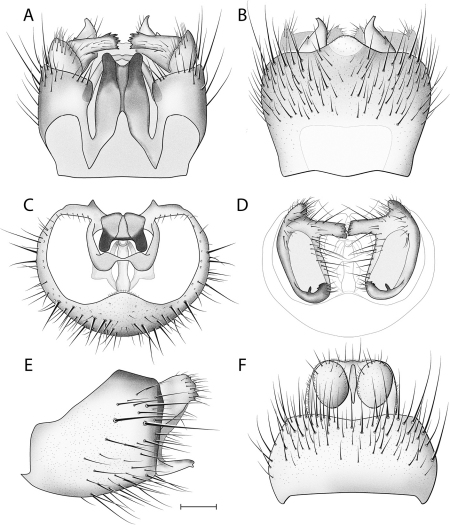
*Acomoptera digitata* sp. n., male genitalia, illustrated: **A** gonopods, dorsal view **B** gonopods, ventral view **C** gonocoxites, with gonostyli removed, posterior view **D** gonostyli, posterior view **E** gonopods, lateral view **F** epandrium, dorsal view. Scale line = 0.1 mm.

#### Comment.

The female specimen was united with males on the basis of similar coloration patterns (e.g., bicolored mediotergite) and having been collected with males at a locality known only for this species.

#### Etymology.

 The species epithet “digitata" is an adjective derived from the Latin word for finger, referring to the adaxial process of the gonostylus.

### Acomoptera echinosa sp. n.

urn:lsid:zoobank.org:act:E046E3C3-A83A-4010-973C-4C5C97528913

http://species-id.net/wiki/Acomoptera_echinosa

**Figure 12.**
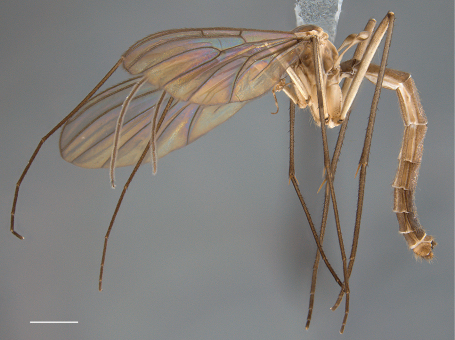
*Acomoptera echinosa* sp. n., habitus, lateral view [691282]. Scale line = 1 mm.

Figs 12 [Fig F13] 14 

#### Type Material.

 Holotype: ♂, “USA: OR: Lincoln Co., Waldport, Malaise trap, 44.4266°N, -124.0513°W, 56masl, 1–15.ix.2010, John D. Pinto CSCA11L044" / “HOLOTYPE 11G099 ♂ *Acomoptera echinosa* Kerr 2011" [red label]. Deposited in CSCA, complete specimen in excellent condition, mounted on gray point.

Paratypes: 1 ♂, “USA: CA: Humboldt Co., Patrick's Point SP, redwood grove behind visitor center, 41°08.11'N, 124°09.28'W, ~10masl, 10.iv.2008-12.ii.2009 P. Kerr, P. Haggard CSCA09L117" [CSCA] 1 ♂, “Canada: B.C., Upper Carmanah Valley, 4.vii–15.vii.1991 N. Winchester, FF.MT4" [48.67°, -124.69°; CASC] 1 ♂, “Canada: B.C., Upper Carmanah Valley, 28.viii–9.ix.1991 N. Winchester, FF.MT1" [48.67°, -124.69°; CASC].

#### Diagnosis.

**Figure 13. F13:**
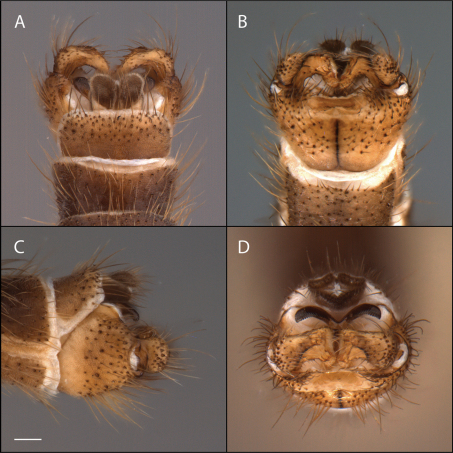
*Acomoptera echinosa* sp. n., male genitalia, images: **A** dorsal view [691278]  **B** ventral view [691279]  **C** lateral view  [691280] **D** posterior view [691281]. Scale line = 0.1 mm.

**Figure 14.**
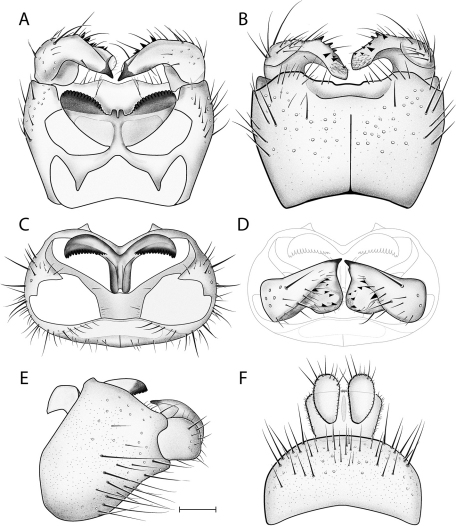
*Acomoptera echinosa* sp. n., male genitalia, illustrated: **A** gonopods, dorsal view [691284]  **B** gonopods, ventral view [691285]  **C** gonocoxites, with gonostyli removed, posterior view **D** gonostyli, posterior view [691287]  **E** gonopods, lateral view [691286]  **F** epandrium, dorsal view [691283]. Scale line = 0.1 mm.

  This species is similar to *Acomoptera nelsoni* sp. n. in that the male gonocoxites display a prominent dorsomedial comb (Fig. 14A) and the outer surface of the gonostyli bear denticulations (Figs 14B, 14D). The gonostyli of both species are also similar in that the dorsoapical margin comes to an acute point and is darker in color than the rest of the gonostylus (Fig. 14D). The gonostyli of *Acomoptera echinosa* sp. n., however, lack denticulations on the inner surface of the gonostyli dorsally (Figs 13A, 14A).

#### Description.

 Male. Body length (n=2): 6.5–6.9 mm (avg = 6.7 mm). Wing length: 4.8–5.7 mm (avg = 5.3 mm).

*Coloration* (Fig. 12). Head brown; palpomeres light brown. Antennal scape light brown, pedicel yellowish, base of first flagellomere yellowish, otherwise flagellomeres brown. Thorax cream-colored to brown; scutum brown to dark brown; dorsocentral areas of scutum defined by lighter brown coloration, scutum setae gold- or golden brown-colored; laterotergite and mediotergite light brown to cream-colored. Legs becoming increasingly brown towards tarsi, coxae cream-colored; femur yellowish or cream-colored, tibia yellowish brown to brown, tarsi brown. Wing hyaline without markings, wing veins light brown; haltere stem and knob cream-colored to light brown. Abdominal segments concolorous, brown, slightly darker posteriorly, with golden brown setae. Terminalia yellowish brown to brown.

*Head.* Ocelli slightly raised; middle ocellus clearly smaller than (approx. 0.25× size of) lateral ocelli, lateral ocellus located approx. width of ocellus or less from eye margin, separated from median ocellus by approx. twice its own diameter or a little more. Eyes with sparse, inconspicuous microsetae, which are approximately as long as width of facet. Face with golden brown setae, longest of which approx. same length as width of face. Antenna and abdomen subequal in length. Palpus approx. 1× width of head (anterior view); length of palpomeres 2 and 3 nearly subequal (palpomere 3 longer); palpomere 4 approx. 6× longer than wide; palpomere 5 approx. 11× longer than wide, subequal to or shorter than combined length of palpomeres 3 and 4.

*Thorax*. Antepronotum bearing setae; remaining thoracic sclerites bare. Tarsal claw usually with one small ventral tooth. Wing venation similar to others in the genus (e.g., *Acomoptera digitata* sp. n., Fig. 8); costal vein extends beyond R_5_, approx. 0.33× distance between R_5_ and M_1_; R_1_, R_5_, and M_1_ with at least some setae on lower surface (ventral M_2_ sometimes with setae, also).

*Male Genitalia* (Figs 13–14). Epandrium approx. 2× wider than long (Fig. 14F). Gonocoxites with developed dorsomedial comb (Figs 14A, 14C). Gonostylus complex, as shown in Figs 13, 14A–B, 14D–E.

Female unknown.

#### Etymology.

The species epithet “echinosa" is an adjective derived from the Greek word meaning hedgehog or sea-urchin, referring to the spiny surface of the gonostylus.

### Acomoptera forculata sp. n.

urn:lsid:zoobank.org:act:3D24FE01-05C3-4A59-9A2E-0E6FB2F342AA

http://species-id.net/wiki/Acomoptera_forculata

**Figure 15.**
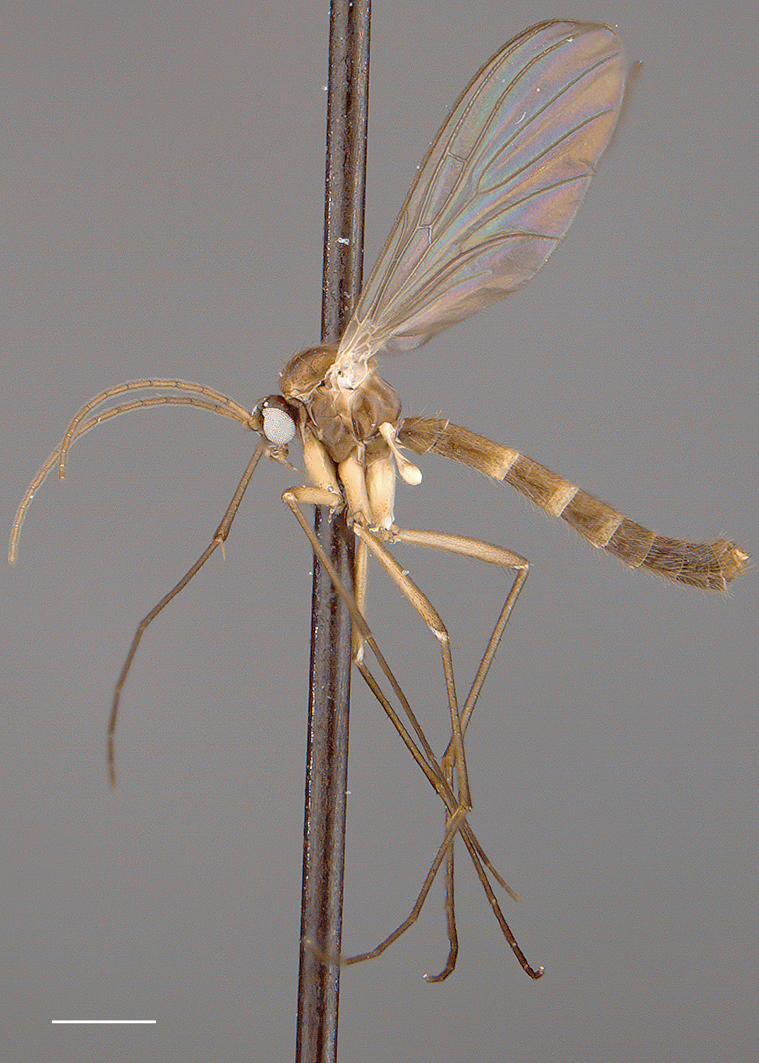
*Acomoptera forculata* sp. n., habitus, lateral view [691292]. Scale line = 1 mm.

Figs 15 [Fig F16] 17 

#### Type Material.

 Holotype: ♂, “7 Mi. E. Griffith, Ont., 31.v.1983, B.E. Cooper" [45.181°, -77.237°] / “HOLOTYPE 11G479 ♂ *Acomoptera forculata* Kerr 2011" [red label]. Deposited in CNC, specimen glued directly to the pin, missing last left antennal segment, right front leg, and left hind leg. Otherwise in excellent condition.

Paratypes: 1 ♂, same data as holotype [CNC]; 1 ♂, “N.S., CBHNt.Pk., Mackenzie Mt., 400m PG639848, 27.V.1984 / birch & fir, B.E. Cooper" [46.738°, -60.650°; CNC]; 1 ♂, same data, except collected 28.V.1984 [CNC]; 1 ♂, same data, except collected 7.VI.1984 [CNC]; 2 ♂, “N.S., CBHNt.Pk., Pleasant Bay PG682873, 6.VI.1984 / mixed forest, B.E. Cooper" [46.738°, -60.650°; CNC]; 1 ♂, “N.S., CBHNt.Pk., North Mt. Bog PG767865, 3.VI.1984, B.E. Cooper" [46.738°, -60.650°; CNC]; 2 ♂♂, “N.S., C.B.N.P., Black Brook, 2–4-VI-1983, Herbs QG035833 6.VI.1984" [46.738°, -60.650°; CNC]; 1 ♂, “Kouchibouguac N.P., N.B., 18.VI.1977, G.A. Calderwood" [46.814°, -64.928°; CNC];

#### Diagnosis.

**Figure 16. F16:**
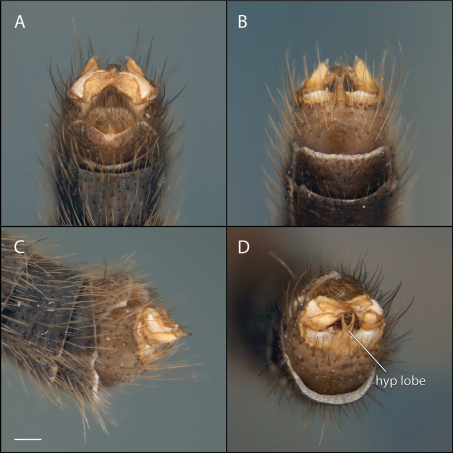
*Acomoptera forculata* sp. n., male genitalia, images: **A** dorsal view [691288] **B** ventral view [691289]  **C** lateral view [691290] **D** posterior view [691291]. Scale line = 0.1 mm. Abbreviations: **hyp lobe** hypandrial lobe.

**Figure 17.**
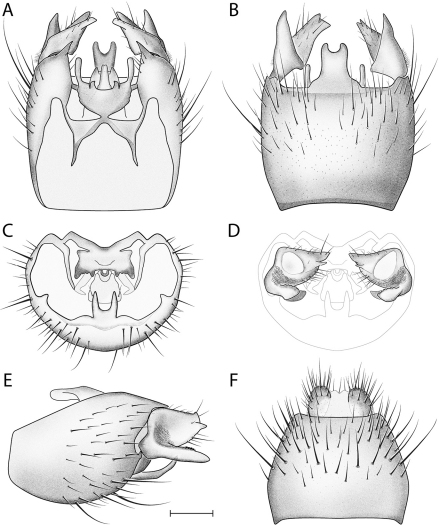
*Acomoptera forculata* sp. n., male genitalia, illustrated: **A** gonopods, dorsal view [691294]  **B** gonopods, ventral view [691295] **C** gonocoxites, with gonostyli removed, posterior view [691297] **D** gonostyli, posterior view [691298] **E** gonopods, lateral view [691296] **F** epandrium, dorsal view [691293]. Scale line = 0.1 mm.

  This species is unique in the genus for having a forked hypandrial lobe (Fig. 16D). The form of the gonostylus (Fig. 17D) is reminiscent of that displayed by *Acomoptera difficilis* (Fig. 5D), but the posterior-projecting ventrolateral process is significantly shorter (Fig. 17E).

#### Description.

 Male. Body length (n=7): 4.5–5.6 mm (avg = 5.0 mm). Wing length: 4.1–4.7 mm (avg = 4.4 mm).

*Coloration* (Fig. 15). Head brown; palpomeres yellowish light brown. Antennal scape brown, pedicel yellowish light brown, base of first flagellomere yellowish light brown, remaining flagellomeres slightly darker. Thorax mostly brown; scutum uniformly brown to dark brown and/or area of acrostichal setae darkened, areas laterad of dorsocentral setae with darker brown markings, scutum setae gold- or golden brown-colored; laterotergite and mediotergite light brown to brown. Legs darkening towards tarsi (although in some cases, hind legs less so), coxae yellowish or cream-colored; femur yellowish light brown, tibia and tarsi light brown to brown. Wing hyaline without markings, wing veins light brown; haltere stem and knob cream-colored to light brown. Abdominal tergites 2–5 brown, with posterior margins yellowish or cream-colored; other segments concolorous brown; abdomen with golden brown setae. Terminalia yellowish brown to brown.

*Head.* Ocelli slightly raised; middle ocellus slightly smaller than (approx. 0.6-0.8× size of) lateral ocelli, lateral ocellus located approx. width of ocellus from eye margin, separated from median ocellus by 2–3× its own diameter. Eyes with sparse, inconspicuous microsetae, which are approximately as long as width of facet. Face with brown setae, longest of which approx. same length as width of face. Antenna approx. 0.75–0.9× length of abdomen. Palpus approx. 0.7× width of head (anterior view); length of palpomeres 2 and 3 nearly subequal (palpomere 3 longer); palpomere 4 approx. 4× longer than wide; palpomere 5 approx. 6× longer than wide, subequal to or shorter than combined length of palpomeres 3 and 4.

*Thorax*. Antepronotum bearing setae; remaining thoracic sclerites bare. Wing venation similar to others in the genus (e.g., *Acomoptera digitata* sp. n., Fig. 8); costal vein extends beyond R_5_, approx. 0.33× distance between R_5_ and M_1_; R_1_, R_5_, and M_1_ with at least some setae on lower surface (although often lacking on M_1_).

*Male Genitalia* (Figs 16–17). Epandrium slightly wider (1.3x) than long (Fig. 17F). Gonocoxites with dorsomedial structure bearing elongated lobes, not developed as dorsomedial comb (Figs 17A, 17C). Hypandrial lobe present, forked (Fig. 16D). Gonostylus complex, as shown in Figs 16, 17A–B, 17D–E.

Female unknown.

#### Etymology.

 The species epithet “forculata" is an arbitrary combination of letters alluding to the prominently forked hypandrial lobe.

### Acomoptera nelsoni sp. n.

urn:lsid:zoobank.org:act:4295F099-F09F-4B04-AAF8-C25F00BA0C49

http://species-id.net/wiki/Acomoptera_nelsoni

**Figure 18.**
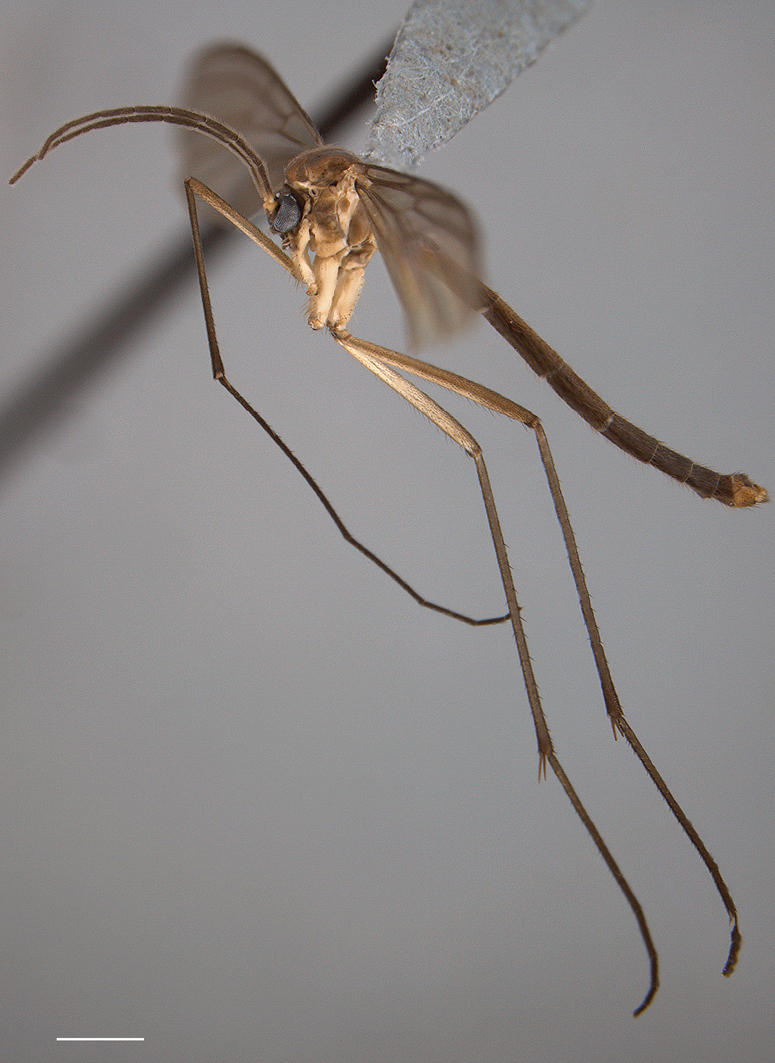
*Acomoptera nelsoni* sp. n., habitus, lateral view [691303]. Scale line = 1 mm.

Figs 18 [Fig F19] 20 

#### Type Material.

 Holotype: ♂, “USA: CA: Humboldt Co., Patrick's Point SP, forest behind visitor center MT#1 (6m), 41°08.11'N, 124°09.28'W, ~10masl, 3.iii-10.iv.2008 P.H.Kerr&P.A.Nelson CSCA08L359" / “HOLOTYPE 10F621 ♂ *Acomoptera nelsoni* Kerr 2011" [red label]. Deposited in CSCA, mounted on gray point, missing ultimate 5 segments of antennae, left front and mid legs, otherwise in good condition. Specimen dissected, male genitalia preserved in DMHF, on card marked “10F621" pinned below specimen.

Paratypes: 3 ♂♂, “Canada: B.C., Upper Carmanah Valley, UTM: 10U CJ 803006, 12–27.viii.1991 N. Winchester, TZ.MT3" [48.67°, -124.69°; CASC]; 1 ♂, “Canada: B.C., Upper Carmanah Valley, 28.viii–9.ix.1991 N. Winchester, TZ.MT4" [48.67°, -124.69°; CASC].

#### Diagnosis.

**Figure 19. F19:**
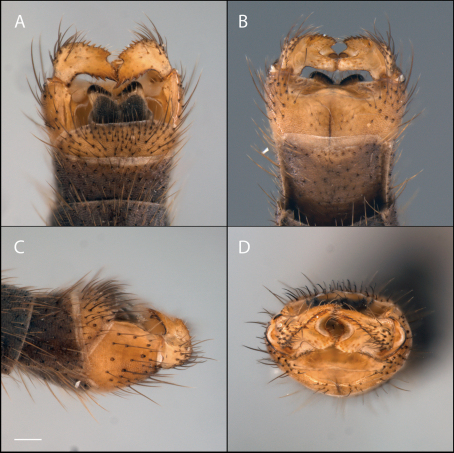
*Acomoptera nelsoni* sp. n., male genitalia, images: **A** dorsal view [691299] **B** ventral view [691300] **C** lateral view  [691301] **D** posterior view [691302]. Scale line = 0.1 mm.

**Figure 20.**
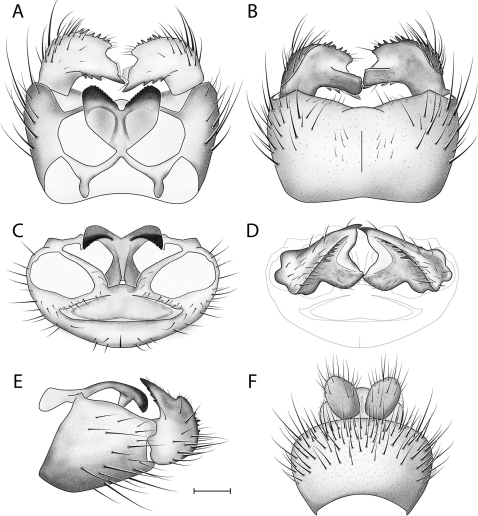
*Acomoptera nelsoni* sp. n., male genitalia, illustrated: **A** gonopods, dorsal view [691305] **B** gonopods, ventral view [691306] **C** gonocoxites, with gonostyli removed, posterior view [691308] **D** gonostyli, posterior view [691309] **E** gonopods, lateral view [691307] **F** epandrium, dorsal view [691304]. Scale line = 0.1 mm.

  The male gonopods of *Acomoptera nelsoni* sp. n. are similar to *Acomoptera echinosa* sp. n., as noted above. The gonostyli of *Acomoptera nelsoni*, however, are unique in having denticulations arranged in rows, which are present on the inner surface of the gonostyli dorsally (Fig. 20A), in addition to the profile of its form in both dorsal and ventral views (Figs 19A–B, 20A–B). The gonocoxal dorsomedial comb is also unique, in being swept back and more narrow than in other *Acomoptera* species that have this structure (Fig. 20A).

#### Description.

 Male. Body length (n=1): 7.1 mm. Wing length (n=1): 6.0 mm.

*Coloration* (Fig. 18). Head brown; palpomeres yellowish darkening to brown distally. Antennal scape light brown, pedicel yellowish, base of first flagellomere yellowish, otherwise flagellomeres brown. Thorax cream-colored to brown; scutum brown to dark brown; darker in areas immediately laterad of dorsocentral setae, scutum setae gold- or golden brown-colored; laterotergite and mediotergite light brown to cream-colored. Legs becoming increasingly brown towards tarsi, coxae cream-colored; femur yellowish or cream-colored, tibia yellowish brown to brown, tarsi brown; hind legs lighter in color. Wing hyaline without markings, wing veins brown; haltere stem cream-colored to light brown, knob brown. Abdominal segments concolorous brown, with brown setae. Terminalia yellowish brown to brown.

*Head.* Ocelli slightly raised; middle ocellus clearly smaller than (approx. .25× size of) lateral ocelli, lateral ocellus located approx. width of ocellus or less from eye margin, separated from median ocellus by approx. twice its own diameter. Eyes with sparse, inconspicuous microsetae, which are approximately as long as width of facet. Face with mostly brown setae, longest of which approx. same length as width of face. Antenna and abdomen elongate (probably) subequal in length. Palpus approx. 1× width of head (anterior view); palpomere 2 clearly shorter than palpomere 3; palpomere 4 approx. 6× longer than wide; palpomere 5 approx. 11× longer than wide, subequal to or shorter than combined length of palpomeres 3 and 4.

*Thorax*. Antepronotum bearing setae; remaining thoracic sclerites bare. Wing venation similar to others in the genus (e.g., *Acomoptera digitata* sp. n., Fig. 8); costal vein extends beyond R_5_, approx. 0.33× distance between R_5_ and M_1_; R_1_, R_5_, and M_1_ with at least some setae on lower surface.

*Male Genitalia* (Figs 19–20). Epandrium approx. 2× wider than long (Fig. 20F). Gonocoxites with developed dorsomedial comb (Figs 20A, 20C). Gonostylus complex, as shown in Figs 19, 20A–B, 20D–E.

Female unknown.

#### Etymology.

 The species is named after Peter A. Nelson of Santa Cruz, CA, long-time mentor and friend. He greatly facilitated the collection of this species and many others in the California North Coast region.

### Acomoptera plexipus

(Garrett)

http://species-id.net/wiki/Acomoptera_plexipus

**Figure 21.**
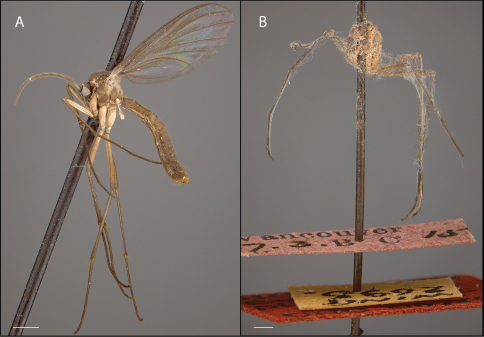
*Acomoptera plexipus*, habitus, lateral view: **A** male [691315] **B** female, holotype [691310]. Scale line = 1 mm.. Scale line = 1 mm.

Figs 21 [Fig F22] 23

Eudicrana plexipus Garrett 1925: 4

#### Type Material.

 Holotype examined: ♀, “Vancouver, B.C., 27.3.15 / wing 2693 / HOLOTYPE, Eudicrana plexipus, Garrett, CNC No7849 / MONOTYPE EUDICRANA PERPLEXUS ♀ <signature> C.B.D. Garrett " [CNC]. Specimen dissected, with female genitalia retained in glass vial pinned below specimen. Specimen in bad condition, covered with fungal spores (Fig. 21B).

#### Material Examined.

1 ♂, “Mt. Thornhill Terrace, B.C., 26-VII 1960, C.H. Mann / along trail in hemlock forest 2500' / 11G478 <dissection card>" [54.532°N, -128.567°W; CNC]; 1 ♂, “Johnston Canyon, 4700' Banff, ALTA., 18 July, 1962, W.R.M. Mason / 11G490 <dissection card>" [51.237°N, -115.855°W; CNC] ; 1 ♂, “Ont., Iroquois Falls, 30.vi.1987, J.R. Vockeroth / Populus-Picea wood; rich undergrowth" [48.768°N, -80.673°W; CNC]; 1 ♂, “N.S., CBHNt.Pk., PG706863, 2.VI.1984, B.E. Cooper" [46.738°N, -60.650°W; CNC]; 1 ♂, “N.S., CBHNt.Pk., Mackenzie Mt., 400m PG639848, 7.VI.1983 / birch & fir, B.E. Cooper" [46.738°N, -60.650°W; CNC]; 1 ♂, “N.B., Victoria Co., 1982, G.R.Parker" [CNC].

#### Diagnosis.

**Figure 22. F22:**
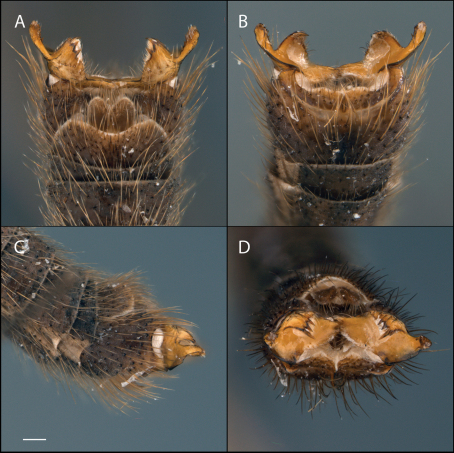
*Acomoptera plexipus*, male genitalia, images: **A** dorsal view [691311] **B** ventral view [691312] **C** lateral view [691313] **D** posterior view [691314]. Scale line = 0.1 mm.

**Figure 23.**
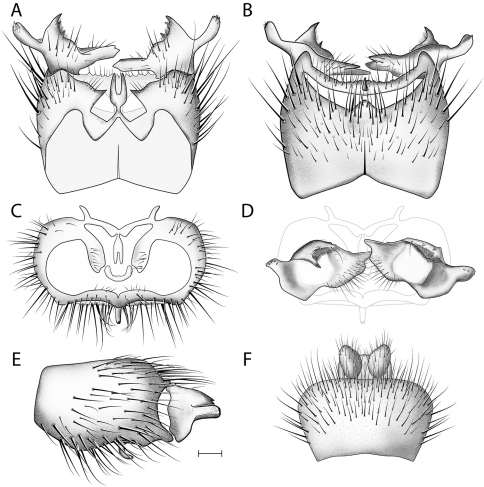
*Acomoptera plexipus*, male genitalia, illustrated: **A** gonopods, dorsal view [691317] **B** gonopods, ventral view [691318] **C** gonocoxites, with gonostyli removed, posterior view [691320] **D** gonostyli, posterior view [691321] **E** gonopods, lateral view [691319] **F** epandrium, dorsal view [691316]. Scale line = 0.1 mm.

  This species is easily distinguished from all other *Acomoptera* in having a short preapical hook arising from the gonocoxites ventromedially (Figs 23B–C, 23E); a narrow, bifid process arising from the gonocoxites dorsomedially (Figs 23A, 23C); and the unique form of the gonostylus (Figs 22, 23A–B, 23D–E).

#### Description.

 Male. Body length (n=4): 5.0–6.1 mm (avg = 5.8 mm). Wing length: 4.7–5.8 mm (avg = 5.3 mm).

*Coloration* (Fig. 21). Head brown; palpomeres yellowish darkening to brown distally or brown throughout. Antennal scape and pedicel brown; base of first flagellomere yellowish, otherwise flagellomeres brown. Thorax mostly brown, with some lighter areas (e.g., mediotergite); scutum brown to dark brown; darker in areas of acrostichal and dorsocentral setae, scutum setae gold- or golden brown-colored. Legs becoming increasingly brown towards tarsi, coxae cream-colored; femur yellowish or cream-colored, tibia yellowish brown to brown, tarsi brown; hind legs lighter in color. Wing hyaline without markings, wing veins brown; haltere cream-colored to light brown. Abdominal segments concolorous brown, with predominantly light, golden setae. Terminalia mostly brown, except gonostyli yellowish.

*Head.* Ocelli slightly raised; middle ocellus approximately same size as lateral ocelli, lateral ocellus located approx. width of ocellus or slightly less from eye margin, separated from median ocellus by approx. twice its own diameter. Eyes with numerous microsetae, longest approx. twice as long as width of facet. Face with mostly brown setae, longest of which approx. same length as width of face. Antenna approximately 2/3rds length of abdomen. Palpus approx. 1× width of head (anterior view); palpomere 2 short, as wide as long, remaining palpomeres longer than wide; palpomere 4 approx. 4× longer than wide; palpomere 5 approx. 7× longer than wide, subequal to or shorter than combined length of palpomeres 3 and 4.

*Thorax*. Antepronotum bearing setae; remaining thoracic sclerites bare. Wing venation similar to others in the genus (e.g., *Acomoptera digitata* sp. n., Fig. 8), may exhibit teratological variations (e.g., loss of R_4_ (Fig. 21; in this specimen, R_4_ lost on left wing but present on right wing)); costal vein extends beyond R_5_, approx. 0.3× distance between R_5_ and M_1_; R_1_, R_5_, and M_1_ with at least some setae on lower surface.

*Male Genitalia* (Figs 22–23). Epandrium approx. 1.7× wider than long (Fig. 23F). Gonocoxites with dorsomedial fork (Figs 23A, 23C) and short ventral hook (Figs 23B–C, 23E). Gonostylus complex, as shown in Figs 22, 23A–B, 23D–E.

Female as described by Vockeroth (1980).

### Acomoptera sinica

Wu & Yang, 1990

Acomoptera sinica  Wu & Yang, 1990: 276

#### Comment. 

Specimens of this species were unavailable for examination. The crude illustrations in the original publication show this species having genitalia that are significantly different from known *Acomoptera* and the description lacks critical additional information. For this reason, the proper generic placement of this species has not been confirmed.

### Acomoptera spinistyla

(Søli, 1993) comb. n.

http://species-id.net/wiki/Acomoptera_spinistyla

Drepanocercus spinistylus  Søli, 1993: 74

#### Material Examined.

2 ♂♂, “SLOVAKIA centr., 1200 m, Polana Biosphere Reserve, Zadná Polana N.N.Res., 6.5.–3.7.2006 Malaise trap, J. Ševčík & J. Roháček leg." [48.66°N, 19.49°E].

*Drepanocercus* was originally defined on the basis of having the cubital fork very near the base of the wing and elongated female cerci (Vockeroth, 1980). *Acomoptera spinistyla* shows neither of these features and in its original placement, prevents a clear distinction between *Drepanocercus* and *Acomoptera*. The spiky gonostyli of *Drepanocercus spinistylus* recall similar conditions found in *Acomoptera* species such as *Acomoptera digitata* (Fig. 11D) and *Acomoptera plexipus* (Fig. 23D). Furthermore, in *Acomoptera spinistyla* and *Acomoptera plexipus*, there is a bifurcate dorsomedial process (Søli, 1993: fig. 2D, Fig. 23A) and a broad gap before the posterior margin of the hypandrium (Søli, 1993: fig. 2B, Fig. 23B). For these reasons, it seems appropriate that this species be transferred to *Acomoptera*. Further study is needed to evaluate the position of *Drepanocercus ensifer*; for now, it remains separated from *Acomoptera* by its original defining characters.

### Acomoptera vockerothi sp. n.

urn:lsid:zoobank.org:act:A2DAD9AC-96F0-4478-B172-A5C4BB21EF14

http://species-id.net/wiki/Acomoptera_vockerothi

**Figure 24.**
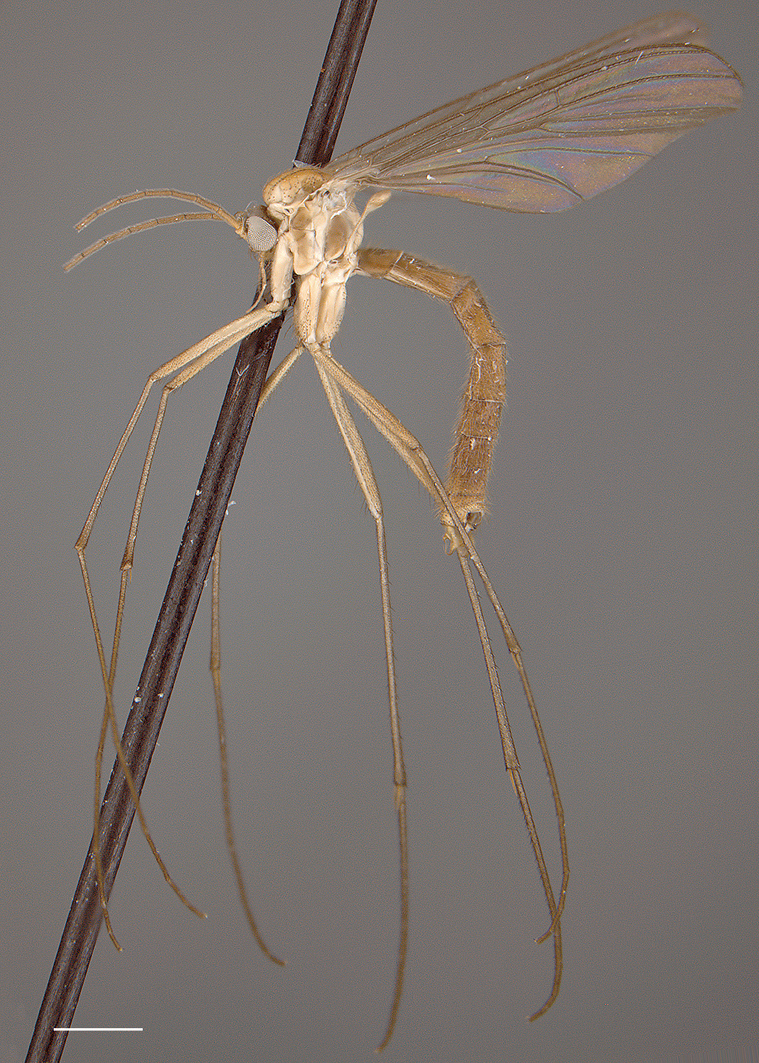
*Acomoptera vockerothi* sp. n., habitus, lateral view [691250]. Scale line = 1 mm.

Figs 24 [Fig F25] 26 

#### Type Material.

 Holotype: ♂, “Can: Manitoba, 2mi., ne. Treesbank, along, Souris R. 11.viii.1993, 49°40'N, 99°36'W, B. Gallaway MT" / “HOLOTYPE 11G627 ♂ *Acomoptera vockerothi* Kerr 2011" [red label]. Deposited in CNC, complete specimen in excellent condition, glued directly to the pin.

Paratypes: **CANADA**: 1 ♂, same data as holotype [CNC]; 4 ♂♂, “Ont., Iroquois Falls, 30.vi.1987, J.R. Vockeroth" [48.768°, -80.673°; CNC]; 1 ♂, “Ont., Iroquois Falls, 7.vii.1987, J.R. Vockeroth / 11G495" [48.768°, -80.673°; CNC]; 1 ♂, “N.Burgess Twp., Lanark Co., ONT., 7.ix.1970, D.M. Wood" [45.010°, -76.359°; CNC]; 1 ♂, “Thwartway Island, St. Lawrence Is. National Park / A. Carter, Aug 27, 1976, Malaise Trap, Code 4529-G" [44.294°, -76.150°; CNC]; 1 ♂, “King Mt., Old Chelsea, QUE., June 16-1960, J.G. Chillcott" [45.489°, -75.864°; CNC]; 1 ♂, “Duncan Lake, Nr. Rupert, Que., 1.IX.1971, J.F.McAlpine" [54.690°, -75.989°; CNC]; 1 ♂, “N.S., CBHNt.Pk., Mackenzie Mt., 300m PG645851, 29.VIII.1983 / Picea Betula woods" [46.738°, -60.650°; CNC]; 1 ♂, “N.S., CBHNt.Pk., Lone Shieling, 300m PG731861, 21.VIII.1983 / Maple forest with fern undergrowth, J.R. Vockeroth" [46.738°, -60.650°; CNC]; 1 ♂, “N.S., S. Harbour, Bch. PG962943, 12.VIII.1983, J.R. Vockeroth" [46.866°, -60.468°; CNC]; **USA**: 1 ♂, “Laurel, MD., 25 May 65, Malaise Trap / 11G482" [39.099°, -76.359°; CNC]; 3 ♂♂, “Laurel, MD., 26 May 65, Malaise Trap" [39.099°, -76.359°; CNC]; 1 ♂, “Highlands, Macon Co., N.C. 3850', 35°3.2'N, 83°11.3'W, June 21, 1958, Jean L. Laffoon" [ISUI]; 1 ♂, “Highlands, Macon Co., N.C. 3850', 35°3.2'N, 83°11.3'W, at light, VII-5-1958, Jean L. Laffoon" [ISUI]; 1 ♂, “Clear Creek, 1 mile so. Highlands, Macon Co., No. Car. 3000', 35°1.5'N, 83°11.5'W, VII-1-1958, J. Laffoon" [ISUI]; 2 ♂♂, “Robin Branch (near Wayah Bald), 4000', Macon Co., No. Car., 35°10.1'N, 83°35.1'W, VII-3-1958, J. Laffoon" [ISUI].

#### Diagnosis.

**Figure 25. F25:**
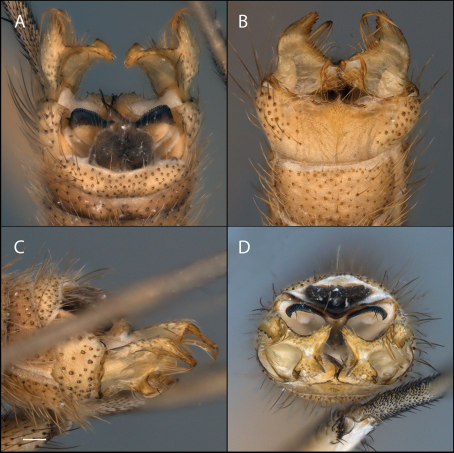
*Acomoptera vockerothi* sp. n., male genitalia, images: **A** dorsal view [691246] **B** ventral view [691247]  **C** lateral view [691248] **D** posterior view [691249]. Scale line = 0.1 mm.

**Figure 26.**
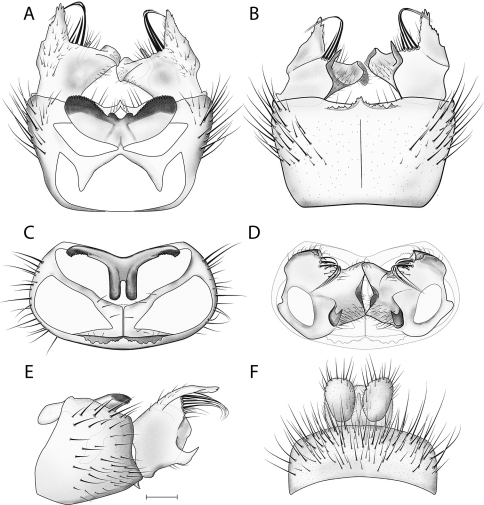
*Acomoptera vockerothi* sp. n., male genitalia, illustrated: **A** gonopods, dorsal view [691252] **B** gonopods, ventral view [691253] **C** gonocoxites, with gonostyli removed, posterior view **D** gonostyli, posterior view [691255] **E** gonopods, lateral view [691254] **F** epandrium, dorsal view [691251]. Scale line = 0.1 mm.

 This species is most similar to *Acomoptera crispa* sp. n. in having similarly-shaped gonostyli that feature a line of long, curved setae near the dorsal margin, as mentioned above. The gonocoxites of *Acomoptera vockerothi* bears a posteromedial denticulate ridge ventrally, however (Figs 25B, 26B). It is also distinguished by having a short, unforked ventromedial gonostylus lobe (Fig. 26E) and the outer dorsal lobe is broadly attached dorsally (Figs 25, 26A–B, 26D–E).

#### Description.

 Male. Body length (n=10): 4.6–6.3 mm (avg = 5.6 mm). Wing length: 4.5–5.3 mm (avg = 5.0 mm).

*Coloration* (Fig. 24). Head brown; palpomeres yellow to yellowish brown. Antennal scape, pedicel, and flagellomeres yellow to yellowish brown, flagellomeres darker than scape and pedicel. Thorax cream-colored to yellowish or light brown; area of scutum bearing acrostichal and dorsocentral setae defined by darker coloration, scutum setae gold- or golden brown-colored. Legs becoming increasingly brown towards tarsi, coxae yellowish or cream-colored; femur yellowish, tibia yellowish brown, tarsi brown. Wing hyaline without markings, wing veins brown; haltere stem and knob yellow to light yellowish brown. Abdominal segments concolorous, yellowish brown to brown, with scattered yellow or golden brown setae. Terminalia yellowish brown.

*Head.* Ocelli slightly raised; middle ocellus approx. same size as lateral ocelli; lateral ocellus located approx. width of ocellus or less from eye margin, separated from median ocellus by approx. twice its own diameter. Eyes with sparse, inconspicuous microsetae, which are approximately as long as width of facet. Face with brown setae, longest of which approx. same length as width of face. Antennal length approx. 0.8× length of abdomen. Palpus approx. 1.0–1.25× width of head (anterior view); length of palpomeres 2 and 3 subequal; palpomere 4 approx. 4.5× longer than wide; palpomere 5 approx. 10× longer than wide, subequal to or longer than combined length of palpomeres 3 and 4.

*Thorax*. Antepronotum bearing setae; remaining thoracic sclerites bare. Wing venation similar to others in the genus (Vockeroth 1980: fig. 3); costal vein extends beyond R_5_, approx. 0.33× distance between R_5_ and M_1_; R_1_, R_5_, M_1_, and M_2_ with at least some setae on lower surface.

*Male Genitalia* (Figs 25–26). Epandrium approx. 3× wider than long. Gonocoxites with dorsomedial comb (Figs 26A). Gonostylus complex, as shown in Figs 25, 26A–B, 25D–E.

Female unknown.

#### Etymology.

 The species is named after J.R. Vockeroth, a remarkably friendly and engaging person, legendary figure in the history of Dipterology, author of the genus, and frequent collector of this species.

## Discussion

Hypotheses of relationship among *Acomoptera* species were not explicitly tested, however shared features of the male genitalia suggest self-evident affiliations. The shared presence of a well-developed dorsomedial gonocoxal comb is an important feature for uniting *Acomoptera crispa*, *Acomoptera echinosa*, *Acomoptera nelsoni*, and *Acomoptera vockerothi* (Figs 2A, 11A, 19A, 25A). All of these species are described for the first time here, and represent a newly-recognized lineage within the genus. Within this group, *Acomoptera crispa* and *Acomoptera vockerothi* exhibit exceptionally similar morphologies of the gonostylus. Overlap in gonostylus form also suggests a close relationship between *Acomoptera echinosa* and *Acomoptera nelsoni*.

A second group appears composed of *Acomoptera forculata*, *Acomoptera plexipus*, and the European species, *Acomoptera difficilis*. These species have a hypandrial lobe in the form of an apical hook or fork and a three-part gonostylus bearing a marginal lobe, whose similarity is especially noticeable when viewed from the lateral perspective (Figs 5E, 17E, 23E). Within this group, the dorsomedial structure of the gonocoxites in *Acomoptera difficilis* and *Acomoptera forculata* is developed in much the same way (Figs 5A, 17A), suggestinga close relationship between these taxa. The position of *Acomoptera digitata* remains unclear.

In light of this newly expanded *Acomoptera* concept, boundaries of sister taxa may be revisited, particularly those of *Drepanocercus*. Chandler (1999) asserted that *Acomoptera spinistyla* is an intermediate between the type species of *Drepanocercus* (*Drepanocercus ensifer* (Garrett 1925)) and *Acomoptera*, based on the intermediate position of the cubital fork. The position of this fork is obscured in some specimens due to the base of CuA_1_ either being weak or obsolete (e.g., Søli 1993: fig. 1), as it is in *Drepanocercus ensifer* (e.g., Vockeroth 1980: fig. 6). Ševčík (2004) notes, however, that there is variation in this character in *Acomoptera spinistyla* and frequently, specimens have CuA_1_ complete, with a clear attachment point to CuA_2_. In such specimens, the fork is approximately at the level of sc-r, just proximad of r-m (Ševčík 2004: fig. 1). The phylogenetic significance of the position of the cubital fork remains unknown in this and other mycetophilid groups, such as in *Tetragoneura* Winnertzand its relatives. For now, however, *Drepanocercus* remains defined on the basis of having the cubital fork very near the base of the wing and elongated female cerci (Vockeroth 1980).

Given the expanded morphological diversity now known to exist within *Acomoptera*, the generic relationships may be addressed in a more comprehensive manner, particularly with respect to *Paratinia* and *Drepanocercus*, and their relation to established members of Gnoristinae and Sciophilinae. This will be the topic of future phylogenetic study.

## Supplementary Material

XML Treatment for Acomoptera

XML Treatment for Acomoptera crispa

XML Treatment for Acomoptera difficilis

XML Treatment for Acomoptera digitata

XML Treatment for Acomoptera echinosa

XML Treatment for Acomoptera forculata

XML Treatment for Acomoptera nelsoni

XML Treatment for Acomoptera plexipus

XML Treatment for Acomoptera sinica

XML Treatment for Acomoptera spinistyla

XML Treatment for Acomoptera vockerothi
